# Microgravity Induces Pelvic Bone Loss through Osteoclastic Activity, Osteocytic Osteolysis, and Osteoblastic Cell Cycle Inhibition by CDKN1a/p21

**DOI:** 10.1371/journal.pone.0061372

**Published:** 2013-04-18

**Authors:** Elizabeth A. Blaber, Natalya Dvorochkin, Chialing Lee, Joshua S. Alwood, Rukhsana Yousuf, Piero Pianetta, Ruth K. Globus, Brendan P. Burns, Eduardo A. C. Almeida

**Affiliations:** 1 Space Biosciences Division, NASA Ames Research Center, Moffett Field, California, United States of America; 2 School of Biotechnology and Biomolecular Sciences, University of New South Wales, Sydney, New South Wales, Australia; 3 Stanford Synchrotron Radiation Lightsource, SLAC National Accelerator Laboratory, Menlo Park, California, United States of America; Institut de Génomique Fonctionnelle de Lyon, France

## Abstract

Bone is a dynamically remodeled tissue that requires gravity-mediated mechanical stimulation for maintenance of mineral content and structure. Homeostasis in bone occurs through a balance in the activities and signaling of osteoclasts, osteoblasts, and osteocytes, as well as proliferation and differentiation of their stem cell progenitors. Microgravity and unloading are known to cause osteoclast-mediated bone resorption; however, we hypothesize that osteocytic osteolysis, and cell cycle arrest during osteogenesis may also contribute to bone loss in space. To test this possibility, we exposed 16-week-old female C57BL/6J mice (n = 8) to microgravity for 15-days on the STS-131 space shuttle mission. Analysis of the pelvis by µCT shows decreases in bone volume fraction (BV/TV) of 6.29%, and bone thickness of 11.91%. TRAP-positive osteoclast-covered trabecular bone surfaces also increased in microgravity by 170% (p = 0.004), indicating osteoclastic bone degeneration. High-resolution X-ray nanoCT studies revealed signs of lacunar osteolysis, including increases in cross-sectional area (+17%, p = 0.022), perimeter (+14%, p = 0.008), and canalicular diameter (+6%, p = 0.037). Expression of matrix metalloproteinases (MMP) 1, 3, and 10 in bone, as measured by RT-qPCR, was also up-regulated in microgravity (+12.94, +2.98 and +16.85 fold respectively, p<0.01), with MMP10 localized to osteocytes, and consistent with induction of osteocytic osteolysis. Furthermore, expression of CDKN1a/p21 in bone increased 3.31 fold (p<0.01), and was localized to osteoblasts, possibly inhibiting the cell cycle during tissue regeneration as well as conferring apoptosis resistance to these cells. Finally the apoptosis inducer Trp53 was down-regulated by −1.54 fold (p<0.01), possibly associated with the quiescent survival-promoting function of CDKN1a/p21. In conclusion, our findings identify the pelvic and femoral region of the mouse skeleton as an active site of rapid bone loss in microgravity, and indicate that this loss is not limited to osteoclastic degradation. Therefore, this study offers new evidence for microgravity-induced osteocytic osteolysis, and CDKN1a/p21-mediated osteogenic cell cycle arrest.

## Introduction

On Earth, at 1 g, mechanical loading of mammalian tissues is an important factor in maintaining tissue health and promoting normal regenerative growth. Forces generated by gravity such as hydrostatic pressure, stretch, and fluid shear are well known to promote normal tissue growth and repair mechanisms. Conversely, during spaceflight, gravity-generated forces are absent and the mechanical stimulation of tissues is greatly diminished. Direct and immediate physiological responses to microgravity-induced mechanical unloading include adaptive losses of bone [1], [2], [3], [4], [5], [6] and muscle mass [7], [8], [9], [10], and alterations in cardiovascular function [11], [12], [13], [14]. Because of practical reasons, most space biological animal research has focused on the short-term effects of spaceflight, and thus our understanding of long-term effects of microgravity exposure is very limited. However, it is likely that over long periods of time mechanical unloading in microgravity may continue to affect tissue regenerative growth and repair, resulting in more widespread degenerative effects. Alternatively, it is also possible that cells and tissues may adapt to microgravity and reach a new homeostatic level appropriate for health maintenance under reduced mechanical load conditions. Since normal tissue repair and regeneration is dependent on the ability of adult stem cells to proliferate and differentiate, we have hypothesized that during long-term spaceflight, the reduction in gravity mechano-stimulation may decrease regenerative proliferation and differentiation of tissue-specific adult stem cells. This in turn may have significant health consequences for multiple tissues throughout the body, including bone.

Specifically for bone tissue, short-duration exposure to microgravity rapidly leads to alterations in bone mineral content, cellular dynamics, and gene expression patterns [15], [16], [17], [18], [19]. This is illustrated by a 1–2% loss of skeletal mineral in weight-bearing bones per month [17], which poses significant risks for long-duration and interplanetary missions. Because bone remodeling homeostasis depends on tightly coupled mineral deposition and resorption [20], [21], uncoupling of these processes in microgravity can cause increased and rapid bone resorption by osteoclasts, and decreased bone formation by osteoblasts [17], [18], [22], [23]. Increased osteoclast bone resorptive activity has been documented within the first several days of spaceflight [1], [17] and may be reduced after a period of transition and adaptation to microgravity. However, of potentially more importance to long duration missions in microgravity is the reported reduction of bone formation by osteoblasts in growing rats [24], [25], [26], although, this result has not been reproduced. In skeletally mature animals, this bone formation arrest may translate into an impairment of regenerative mechanisms, and therefore may be an important long-term factor in spaceflight. Short of conducting long-term experiments with rodents in space, analysis of the early cell cycle arrest signaling pathways in osteogenic bone cells after short-term spaceflight is the most practical current approach to predict the long-term degenerative effects of microgravity.

To perform this analysis we focused on the activation of cell cycle arrest and apoptosis pathways such as those mediated by the tumor and growth suppressor, p53, and the cell cycle inhibitor, CDKN1a/p21. P53 levels are reported to increase in the muscle of hindlimb-unloaded animals [27], potentially leading to cell cycle arrest and/or apoptosis. Furthermore, p53 knockout mice have preserved trabecular bone volume following hindlimb-unloading [28] and generally, osteopetrotic bone, suggesting a role for the p53-signaling pathway in the regulation of bone formation and degradation [29], [30]. More remarkably, the CDKN1a/p21 knockout mouse has a viable phenotype with strong tissue regenerative abilities, such as limb re-growth, otherwise unheard of in adult mammals [31]. CDKN1a/p21 can be activated by environmental conditions such as oxidative stress and radiation, or be part of normal cell cycle arrest mechanisms during cell differentiation. This phenotype suggests CDKN1a/p21 up-regulation in microgravity could explain broad regenerative deficits in space.

Recent studies have highlighted the potential role of osteocytes, terminally differentiated osteoblasts embedded within the mineralized matrix, in the bone remodeling process, including in osteolysis of the lacunar surface for maintenance of calcium homeostasis [32], [33]. Results from light and electron microscopy of rat bone in a 22-day spaceflight experiment conducted in 1977 indicated the appearance of lacunae changed to a “wide” morphology that could contribute to bone loss in space [34]. Recently, it was found that osteocytes acquired a more rounded shape after 91-days in microgravity during the Mice Drawer System experiment on ISS, albeit no statistical significance was obtained due to low n, and the authors did not conclude that osteocytic degradation of the lacunae surface was occurring [35]. On the other hand, osteocytic osteolysis was shown to be present in lactating mice but not activated in animals that were hindlimb unloaded for 4 weeks [36], leaving the potential role and importance of osteocytic osteolysis during spaceflight an open question.

To address the limited understanding of cellular and molecular mechanisms underlying bone degeneration in space following the initial osteoclastic activation, we focused on the processes related to osteoblastic and osteocytic activities. We hypothesized that decreases in bone formation during spaceflight may be mediated by an inability of cells in osteogenic lineages to proliferate and differentiate normally due to reduced mechanical stimuli in microgravity. Finally, we also hypothesized that reduced mechanostimulation of osteocytes in microgravity may signal the activation of osteocytic osteolysis of lacunar surfaces, with accompanying activation of matrix degradation pathways. Our spaceflight results presented here, suggest that the bone degenerative effects of microgravity in skeletally mature mice go beyond rapid osteoclastic activation, affecting also cell cycle regulation in the osteogenic lineage, and inducing osteocytic osteolysis.

## Results

### Body Mass of Animals Pre- and Post-Flight

All flight and ground control mice were observed to be in good condition by veterinary examination immediately post-landing. Noticeably, the posture of space-flown mouse tails after landing was vertical, indicating increased vestibular function in this group. The average weight loss of space-flown mice was 2.0 g (−9.1%, SD = 2.1 g, p = 0.013) significantly lower than pre-flight values, consistent with tissue atrophy in microgravity, while synchronous ground control mice only lost an average of 0.7 g (−3.4%, SD = 0.9 g, p = 0.096) of their body weight relative to the beginning of spaceflight. The difference in body weight loss averages for flight versus ground controls was not statistically significant (p = 0.143). Flight habitats showed utilization of resources on average of 10% less food, and 24% less water than synchronous ground controls. However, food and water utilization does not account for actual consumption by mice, because differences may occur in food bar and water losses due to crumbling and spillage in microgravity versus 1 g.

### Spaceflight Causes Osteoclast and Osteocyte-Mediated Bone Loss in the Pelvic Region of Mice

**Figure 1:**
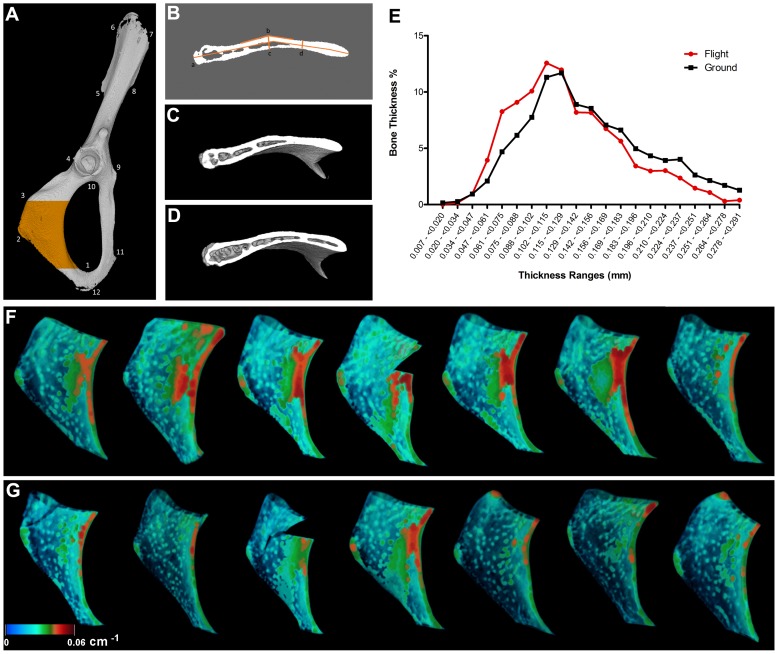
Micro-Computed Tomography (μCT) Analysis of Spaceflown Ischium. The ischium of the pelvis, shown in orange (**A**), was analyzed using μCT (720 slices = 4.89 mm). The anatomical markers used for μCT analysis were (1) caudal apex of obturator foramen, (2) dorsal-most point of the ventral ramus of ischium, and (3) the ischial tuberosity (**A**) (For full details on anatomical markers see [74]). The ischial cross-sectional geometry (**B**) was analyzed by length (a), the width at the midpoint (b) and at 1/3 distance from the obturator foramen (c); and the bend angle (d). Flight samples (**D**) exhibited a more open cross-sectional geometry compared to the ground control (**C**), indicating a possible reduction in the pull force applied to the bone. Ground control samples (**F**) also exhibited greater thickness (orange/red) then the flight samples (**G**), indicating a reduction in overall bone thickness in spaceflight samples.

To determine if bone loss occurred in microgravity, we examined the bone structure and mineral density of the ischial region of the pelvis using micro-computed tomography (µCT). Specifically, we investigated the region from the caudal apex of the obturator foramen through to the ischial tuberosity (Fig. 1A) and found that spaceflight resulted in a 6.23% decrease (p = 0.005) in bone volume fraction (BV/TV) and 11.91% decrease (p = 0.002) in overall bone thickness (Th) (Table 1). No difference was observed in bone mineral density and total bone volumes measured (TV and BV) (Table 1). However, comparison of ischial structural thickness of spaceflight versus ground control animals revealed that bone from flight animals not only had a reduced overall thickness (Fig. 1F and G), but also showed changes in ischial shape localized to the surface of the descending ramus of ischium along the posterior margin of the obturator foramen (Fig. 1B). Analysis of cross-sectional slices revealed that the flight samples had similar widths at two measured points (b/c ratio of 1.09, Fig. 1D, Table 2), and a mean bend angle of 167° (deviating 13° from linear, or less bent), compared to a width ratio of 1.39 in ground control samples and a bend angle of 160° (deviating 20° from linear, or more bent) (Fig. 1C, Table 2), indicating that ischial geometry changes in response to spaceflight. Furthermore, when we analyzed the thickness distribution of ischial cross-sectional slices, we found that flight animals exhibited a higher percentage of slices in the 0.007–0.128 mm range and a lower percentage of slices in the 0.129–0.291 mm range compared to ground controls (Fig. 1E). The shift in thickness distribution to the left in flight animals indicates an overall decrease in cross-sectional thickness throughout the ischium.

**Table 1:** Morphometric Parameters Investigated in µCT Analysis (n = 7).

Parameter	Ground Control	Spaceflight	P-value	% Difference	
**Total Volume (TV), mm^3^**	3.3±0.4	3.1±0.5	0.515		
**Bone Volume (BV), mm^3^**	2.3±0.3	2.3±0.3	0.095		
**Bone Volume Fraction (BV/TV), %**	77.7±0.9	72.8±4*	0.005		−6.29
**Average Bone Thickness, mm**	0.15±0.008	0.13±0.01*	0.002		−11.91
**Linear Attenuation Coefficient**	5.1E−2±2.7E−3	5.1E−2±1.5E−3	0.751		

* Significantly less than ground control, p<0.05. Means are reported ± standard deviation.

**Table 2:** Measurements of the Ischial Cross-Section Cut at the Widest Point of the Descending Ramus (n = 7). Refer to Fig. 1.

Parameter	Ground Control	Flight	P-value
**Length, mm**	3.2±0.1	3.2±0.1	0.350
**Width at point ** ***b*** **, mm**	0.24±0.03	0.20±0.03*	0.029
**Width at point ** ***c*** **, mm**	0.17±0.02	0.18±0.03	0.336
***b/c*** ** ratio**	1.37	1.09*	0.000
**Bend angle,°**	159.7±2.5	166.7±3.43^#^	0.001

* Significantly less than ground control, p<0.05,^ #^Significantly less than ground control, p<0.01. Means are reported ± standard deviation.

**Figure 2:**
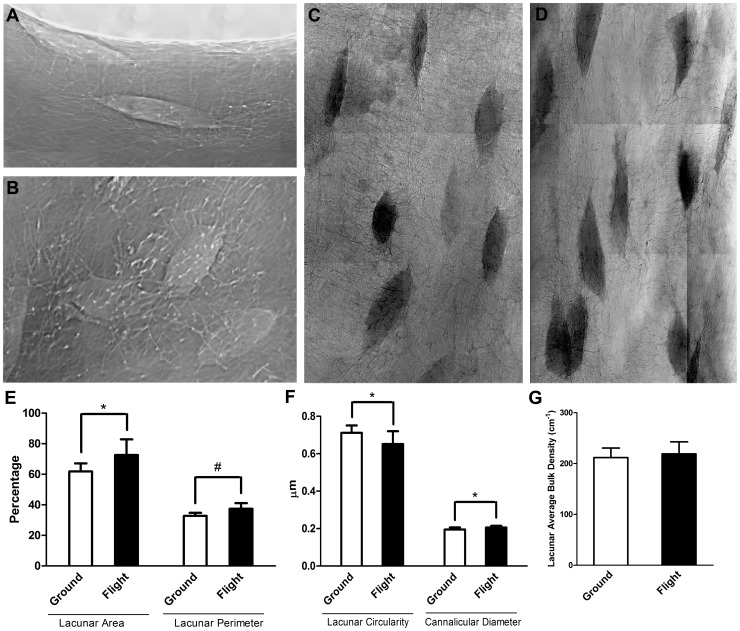
Nano-Computed Tomography Analysis of Lacunar Enlargement Following Spaceflight. Figures 2**A **and** 2B** show X-ray phase contrast images of osteocytes viewed laterally and from the top, respectively and illustrate the flattened shape of osteocytic lacunae. For quantification of lacunar size we used X-Ray absorption images of lateral views of lacunae in ischial cortical bone from ground control (**C**) and spaceflight (**D**) animals (n = 7). We observed a 17% increase in lacunae area and a 14% increase in lacunae perimeter of flight animals compared to ground controls (**E**). We also found a 13% increase in lacunae canalicular diameter and a 9% decrease in lacunae circularity of flight animals compared to ground controls (**F**). However, bulk density analysis showed no statistical difference between flight and ground control animals that is in agreement with µCT analysis (**G**). *indicates p<0.05, #indicates p<0.01.

In order to study nanoscale aspects of bone loss following spaceflight, we used transmission x-ray microscopy to perform 40 nm resolution nano-computed tomography (nanoCT). No difference was found in bulk density of cortical bone between the flight and ground controls (Fig. 2G), however, flight samples exhibited an enlargement in lacunae of 17% in cross sectional area (p = 0.022) and a 14% increase in lacunae perimeter (p = 0.008) when compared to ground controls (Fig. 2C–D, E). In addition, lacunar circularity decreased in spaceflight by 9% (p = 0.037) indicating an increase in irregularly-shaped or elongated lacunae (Fig. 2A–B, F). We also measured canalicular diameters in a 15 µm perilacunar region and found an increase of 6% in spaceflight samples relative to ground controls (p = 0.037, Fig. 2F), indicating that the observed lacunar enlargement may extend throughout these networks.

**Figure 3:**
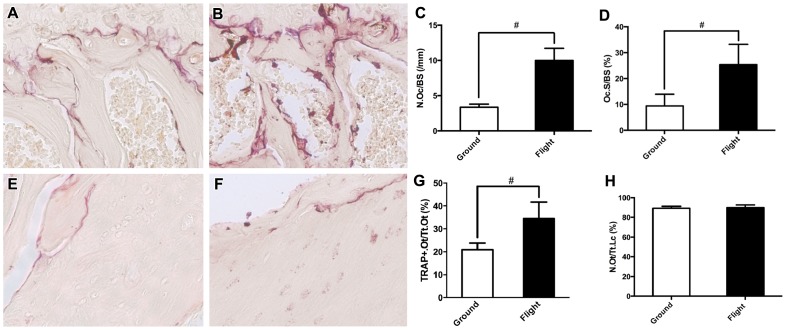
TRAP Staining of Osteoclast and Osteocyte-Mediated Bone Resorption Following Spaceflight. Panel **A** displays a representative cancellous region near the femoral head growth plate (40×) from ground control mice, which is mostly free of TRAP-positive osteoclasts, whilst **B** displays a similar region from flight animals with numerous TRAP-positive osteoclasts. Analysis of osteoclastic activity in the trabecular region below the femoral head of the femur showed an increase in osteoclast numbers in the bone surface of the growth plate of flight samples compared to ground controls (9.99 Oc/mm and 3.36 Oc/mm respectively) (**C**). The bone surface covered by osteoclasts was also increased in flight animals compared to ground controls (25.40% and 9.99% respectively, **D**). The number of TRAP-positive osteocytes in cortical bone from the femoral shaft proximal to the femoral head was increased in response to spaceflight (**E**) compared to ground controls (**D**) (34.43% and 20.94% respectively, **F**). However, we found no differences in the number of empty lacunae in cortical bone between flight and ground controls (10.6% and 10.0% empty lacunae respectively, **H**). * indicates p<0.05, # indicates p<0.01.

To characterize osteoclast bone resorbing activity during spaceflight, we used TRAP staining on sections of the proximal femur. We found a 197% increase in osteoclast numbers in the bone surface of the trabecular region below the femoral head growth plate of flight samples (9.99 Oc/mm) compared to ground controls (3.36 Oc/mm) (p = 0.001, Fig. 3A–C). The bone surface covered by osteoclasts also increased by 154% in microgravity (25.4% in spaceflight versus 9.99% in ground controls, p = 0.004 Fig. 3D). Results for spaceflight induction of all TRAP-positive osteoclasts, independent of number of nuclei, were comparable. Interestingly, we also found an increase in the ratio of TRAP-positive osteocytes to total osteocytes in the femoral shaft of flight samples compared to ground controls (34.43% and 20.94% respectively, p = 0.004, Fig. 3D–G). To determine if osteocyte survival was altered by microgravity we measured the number of empty lacunae in cortical bone from the proximal femur and found no differences between ground and flight samples (10.6% and 10.0% empty lacunae respectively, p = 0.680, Fig. 3H). Pyknotic nuclei were occasionally present in tissue surrounding bone, but were non-detectable in osteocytes. These results indicate roles for both osteoclastic and osteocytic bone degradation in microgravity.

### Spaceflight Induces MMP-Mediated Extracellular Matrix Remodeling in Bone

**Figure 4:**
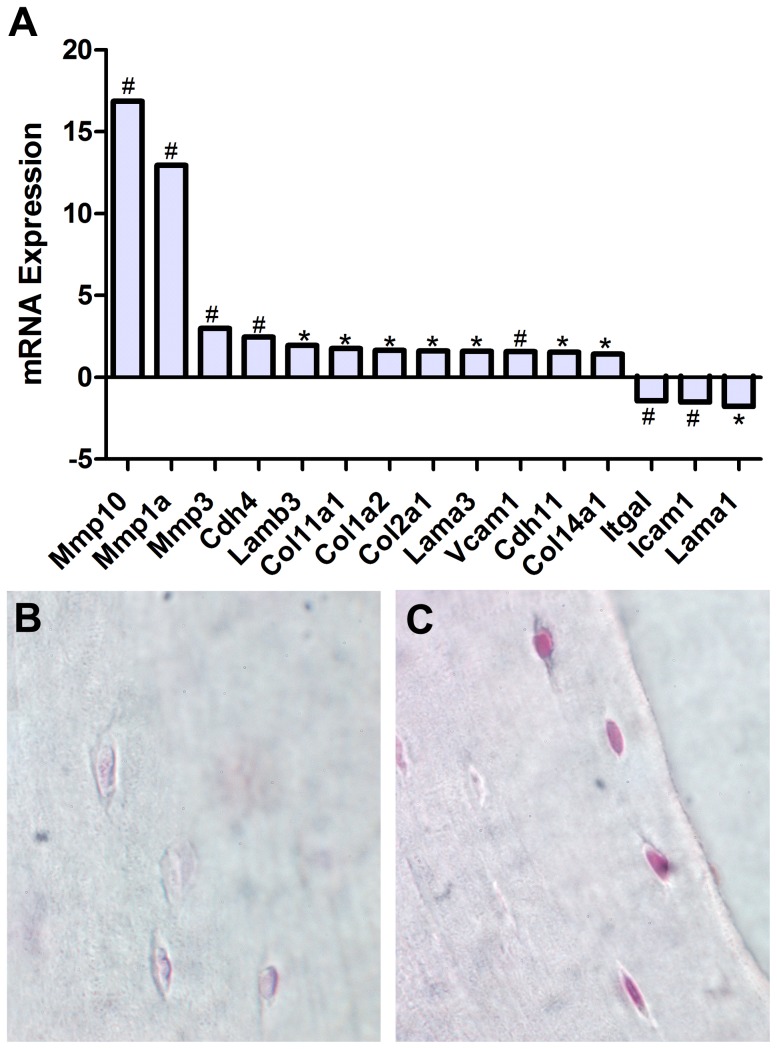
Spaceflight Causes Up-Regulation of Matrix Degradation Molecules. RT-PCR analysis of ilium revealed significant up-regulation of matrix degradation molecules MMP1a, MMP3, and MMP10 as well as small changes in a number of extracellular matrix molecules in flight samples compared to ground controls (**A**). Immunohistochemical analysis localized over-expression of MMP10 to osteocytes in the shaft of the proximal femur in flight samples (**C**) but not in ground controls (**B**), indicating a role for osteocytes in lacunae degradation. * indicates p<0.05, # indicates p<0.01.

Among bone extracellular matrix related genes analyzed using RT-qPCR arrays we found small but statistically significant alterations in mRNA levels of collagens, and laminin subunits, but not in fibronectin (Fig. 4A, Table 3). Among matrix metalloproteinases, MMP 1, 3, and 10 showed significant up-regulation in flight samples compared to ground controls (12.94, 2.98, and 16.85 fold respectively, p<0.05), while MMP inhibitors, TIMP 1, 2, and 3, displayed no statistically significant change in gene expression levels (Table 3). Several cell adhesion molecules; specifically Cdh4, Cdh11, and Vcam1 were found to be up-regulated (2.46, 1.53, and 1.57 fold respectively, p<0.05), while Itgal and Icam1 were found to be down-regulated (1.45 and 1.52 fold respectively, p<0.05) (Fig. 4A, Table 3). These results indicate that spaceflight promotes bone extracellular matrix degradation, while overall increase in expression of collagen, Tuft1, Sparc, and Phex (Table 3), may be related to increased bone mineralization following degradation.

**Table 3:** mRNA Gene Expression Levels Altered Following Spaceflight.

Gene Symbol	Gene Name	P-value	Log^2^ Fold Change
Akt1	Thymoma viral proto-oncogene 1	0.32367	−1.164
Akt2	Thymoma viral proto-oncogene 2	0.65125	1.048
Akt3	Thymoma viral proto-oncogene 3 (PKB)	0.00432	−1.324
Anapc2	Anaphase promoting complex subunit 2	0.00037	1.419
Bmp4	Bone morphogenetic protein 4	0.01240	−1.990
Btg2	B-cell translocation gene 2, anti-proliferative	0.00076	−1.498
Ccnb2	Cyclin B2	0.02484	1.629
Cdc25c	Cell division cycle 25 homolog C (S. pombe)	0.00008	1.551
Cdh11	Cadherin 11	0.02569	1.533
Cdh4	Cadherin 4	0.00279	2.463
Cdk2	Cyclin-dependent kinase 2	0.87024	−1.052
Cdk4	Cyclin-dependent kinase 4	0.90747	1.038
Cdkn1a	Cyclin-dependent kinase inhibitor 1A (p21)	0.000003	3.307
Cdkn1b	Cyclin-dependent kinase inhibitor 1B	0.39548	−1.121
Cdkn2b	Cyclin-dependent kinase inhibitor 2B (p15)	0.04005	1.409
Col11a1	Collagen, type XI, alpha 1	0.01885	1.752
Col14a1	Collagen, type XIV, alpha 1	0.01371	1.407
Col1a2	Collagen, type I, alpha 2	0.02999	1.642
Col2a1	Collagen, type II, alpha 1	0.02863	1.599
Cradd	CASP2 and RIPK1 domain containing adaptor with death domain	0.00215	1.619
Csf3	Colony stimulating factor 3 (granulocyte)	0.02020	3.717
Ctgf	Connective tissue growth factor	0.02194	1.412
Cul3	Cullin 3	0.00346	1.412
Dapk1	Death associated protein kinase 1	0.00696	1.525
Egr1	Early growth response 1	0.00938	−2.296
Eif4e	Eukaryotic translation initiation factor 4E	0.03265	−1.404
Eif4ebp1	Eukaryotic translation initiation factor 4E binding protein 1	0.01475	1.547
Fbxo3	F-box protein 3	0.00017	1.483
Fbxo31	F-box protein 31	0.00004	1.739
Fbxo4	F-box protein 4	0.00040	1.870
Fgf1	Fibroblast growth factor 1	0.00262	1.528
Fgf2	Fibroblast growth factor 2	0.37555	−1.098
Fgf3	Fibroblast growth factor 3	0.00074	−1.699
Fgfr1	Fibroblast growth factor receptor 1	0.01634	1.574
Fgfr2	Fibroblast growth factor receptor 2	0.01981	1.502
Fn1	Fibronectin 1	0.73376	1.037
Fos	FBJ osteosarcoma oncogene	0.04676	−2.366
Gdf10	Growth differentiation factor 10	0.00736	−1.425
Hecw2	HECT, C2 and WW domain containing E3 ubiquitin protein ligase 2	0.00098	2.246
Icam1	Intercellular adhesion molecule 1	0.00001	−1.523
Itgal	Integrin alpha L	0.00263	−1.445
Kras	V-Ki-ras2 Kirsten rat sarcoma viral oncogene homolog	0.04753	−1.243
Lama1	Laminin, alpha 1	0.01601	−1.774
Lama3	Laminin, alpha 3	0.04620	1.582
Lamb3	Laminin, beta 3	0.01095	1.947
Mapk10	Mitogen-activated protein kinase 10	0.00003	3.807
Mapk11	Mitogen-activated protein kinase 11	0.02050	−1.541
Mapk12	Mitogen-activated protein kinase 12	0.00129	−1.431
Mdm2	Transformed mouse 3T3 cell double minute 2	0.00241	1.410
Mmp10	Matrix metallopeptidase 10	0.00045	16.849
Mmp1a	Matrix metallopeptidase 1a	0.00103	12.939
Mmp3	Matrix metallopeptidase 3	0.00119	2.977
Mos	Moloney sarcoma oncogene	0.00322	−4.178
Mul1	Mitochondrial ubiquitin ligase activator of NFKB 1	0.00004	1.644
Myod1	Myogenic differentiation 1	0.01556	−1.607
Nf1	Neurofibromatosis 1	0.01834	−1.445
Nfatc4	Nuclear factor of activated T-cells, cytoplasmic, calcineurin-dependent 4	0.00017	−1.980
NFκB1	Nuclear factor of kappa light polypeptide gene enhancer in B-cells 1, p105	0.04514	−1.271
NFκBIa	Nuclear factor of kappa light polypeptide gene enhancer in B-cells inhibitor, alpha	0.00016	2.027
Phex	Phosphate regulating gene with homologies to endopeptidases on the X chromosome	0.01638	1.435
Pik3ca	Phosphatidylinositol 3-kinase, catalytic, alpha polypeptide	0.00563	−1.386
Pik3cg	Phosphoinositide-3-kinase, catalytic, gamma polypeptide	0.59288	−1.071
Pik3r1	Phosphatidylinositol 3-kinase, regulatory subunit, polypeptide 1	0.31547	−1.220
Pik3r2	Phosphatidylinositol 3-kinase, regulatory subunit, polypeptide 2	0.87773	−1.123
Rps6kb1	Ribosomal protein S6 kinase, polypeptide 1	0.00023	−2.051
Sos1	Son of sevenless homolog 1 (Drosophila)	0.00007	−2.511
Sox9	SRY-box containing gene 9	0.00262	−2.279
Sparc	Secreted acidic cysteine rich glycoprotein	0.00694	1.562
Stat1	Signal transducer and activator of transcription 1	0.00203	−1.565
Tgfb2	Transforming growth factor, beta 2	0.00330	−1.586
Timp1	Tissue inhibitor of metalloproteinase 1	0.63317	1.073
Timp2	Tissue inhibitor of metalloproteinase 2	0.45564	1.072
Timp3	Tissue inhibitor of metalloproteinase 3	0.18863	−1.734
Traf1	Tnf receptor-associated factor 1	0.00018	−2.482
Trp53	Transformation related protein 53	0.00416	−1.535
Trp73	Transformation related protein 73	0.19889	2.019
Tuft1	Tuftelin 1	0.00816	1.486
Twist1	Twist homolog 1 (Drosophila)	0.00550	1.799
Ube2e3	Ubiquitin-conjugating enzyme E2E3, UBC4/5 homolog (yeast)	0.00025	1.847
Vcam1	Vascular cell adhesion molecule 1	0.00876	1.568
Vdr	Vitamin D receptor	0.00003	2.804

To determine the cellular location of MMP10 we conducted immunohistochemical analysis on paraffin embedded sections of the proximal femur. Results showed MMP10-positive staining in osteoblasts along the periosteal surface of the proximal femur in both flight and ground controls. However, osteocytes embedded in the bone matrix exhibited strong MMP10-positive staining in flight samples (Fig. 4C) but not in ground (Fig. 4B).

### Spaceflight Suppresses Pro-Osteogenic Growth and Proliferation Gene Expression in Bone

**Figure 5:**
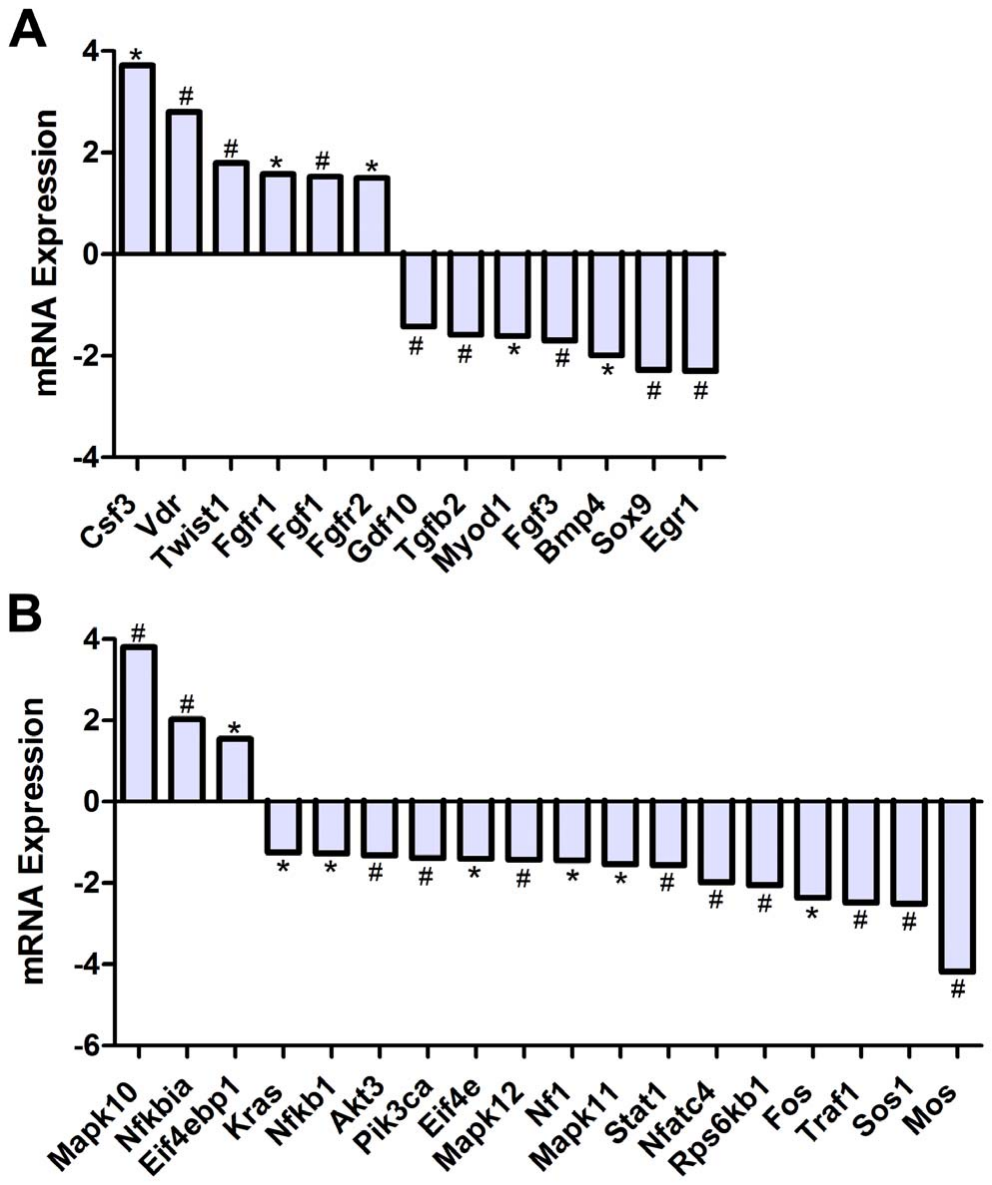
Spaceflight Alters mRNA Expression of Genes Associated with Osteogenic Growth and Mitogenic Signal Transduction Pathways. RT-PCR analysis of revealed altered expression levels of key genes involved in osteogenic growth and proliferation including growth factors, Bmp4 and Tgfβ2, and transcription factors Vdr and Sox9 (A). Analysis of key mitogenic signal transduction pathways revealed alterations in gene expression of the MAPK pathway, whilst Pi3K and Akt signaling molecules were not changed statistically. We also observed significant up-regulation of the NFκB inhibitor, NFκBIa/IκBα (B).

To further study the effects of spaceflight in reducing bone formation, we investigated the expression of genes involved in promoting the cellular growth, proliferation, and differentiation of the osteoprogenitor lineage. A number of growth factors studied showed altered gene expression levels by qPCR, including Bmp-4, Tgf-β2, Gdf10, and Csf3 (−1.99, −1.59, −1.43, 3.72 fold, p<0.05) (Fig. 5A, Table 3). Furthermore, we found several genes associated with cell growth and differentiation that were altered, including Egr1, Myod1, and Fgf3 which were down-regulated (−2.30, −1.61, and −1.7 fold respectively, p<0.05), while Twist1 and Fgf1 were up-regulated (1.80 and 1.53 fold respectively, p<0.01) (Fig. 5A, Table 3). Results also indicate the alteration in gene expression of two transcription factors, Vitamin D receptor (Vdr) and Sox9, which exhibited a 2.80 fold up-regulation and 2.28 fold down-regulation respectively (Fig. 5A, Table 3).

### Spaceflight Induces CDKN1a/p21 but Not Trp53/p53 Expression in Bone

**Figure 6:**
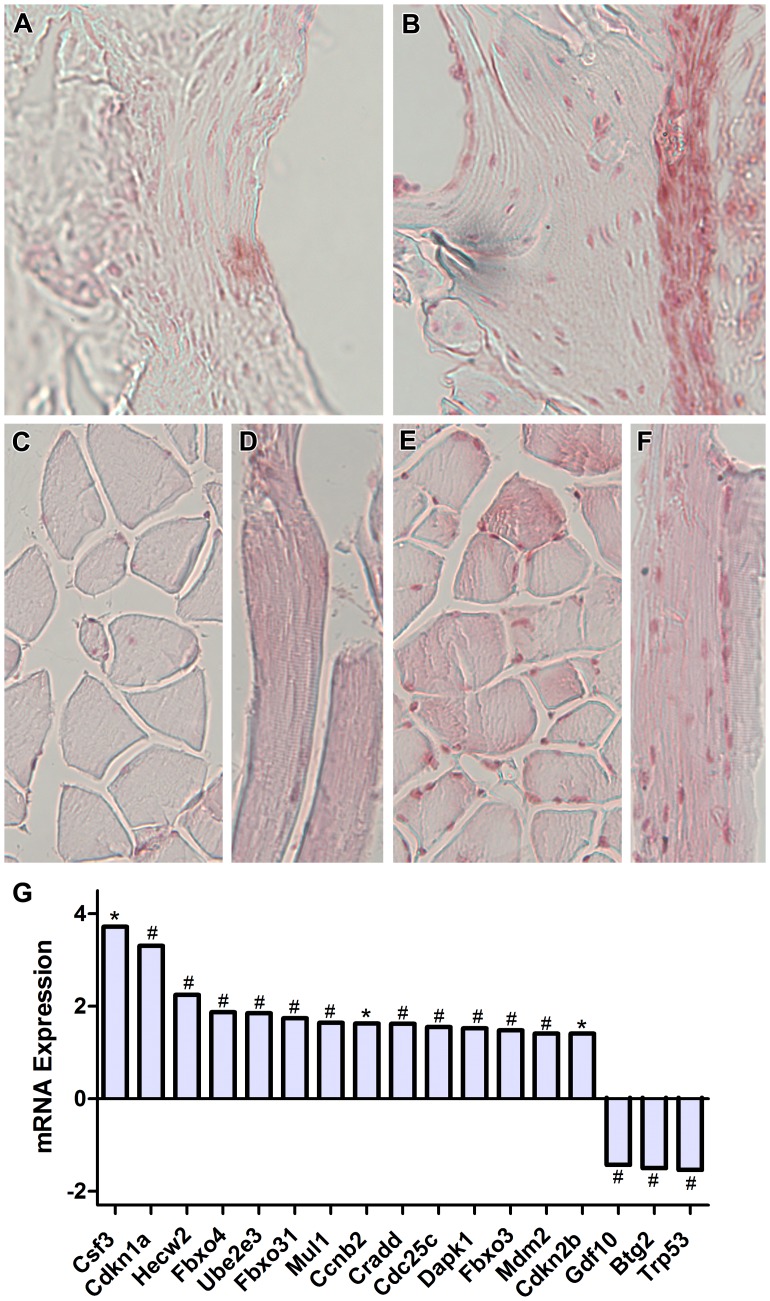
Spaceflight Causes Overexpression of the Cell Cycle Arrest Molecule, p21, Independently of p53 Activation. RT-PCR analysis revealed significant alterations in many cell cycle molecules including a 3.31 fold up-regulation of p21 and down-regulation of p53 (**G**). Immunohistochemical analysis localized this overexpression of p21 to osteoblasts along the periosteal surface of the proximal femur (**A**, ground control, **B**, flight). Interestingly, we also observed p21-positive nuclei in cross-sections and longitudinal sections of muscle fibers adjacent to the femur (**C–D**, ground control, **E–F**, flight). *indicates p<0.05, # indicates p<0.01.

In addition to the effects we observed on osteogenesis promoting gene expression, we hypothesized that bone loss in space may also be mediated by alterations in intracellular signaling cascades resulting in activation of the p53/p21 signaling pathways that promote cell cycle arrest and apoptosis. Analysis of spaceflight samples by qPCR showed that mRNA expression levels of tumor suppressor gene p53 (Trp/p53) were down-regulated 1.54 fold while CDKN1a/p21 exhibited a 3.31 fold up-regulation in response to exposure to spaceflight (p<0.01) (Fig. 6G). The expression of Mdm2, a negative regulator of p53, was also increased, indicating suppression of the p53 apoptotic pathway (Fig. 6G, Table 3). Several genes associated with cell cycle regulation were also altered in flight samples including cell cycle arrest related genes Fbxo4, Fbxo31, Btg2, and Cdkn2b (1.87, 1.74, −1.50, and 1.41 fold respectively, p<0.05), and cell cycle related genes cyclin B2, Cdc25c, Mul1, Gdf10, and Csf3 (1.63, 1.55, 1.64, −1.43 and 3.72 fold respectively, p<0.05) (Fig. 6G, Table 3). Several genes related to the induction of apoptosis were also altered including, Cradd, Dapk1, Fbxo3, which were up-regulated in flight samples (1.62, 1.53, and 1.48 fold respectively, p<0.01) (Fig. 6G). Hecw2, which acts to stabilize p73, was also up-regulated in flight samples (2.25, p<0.01), as was p73, albeit not statistically significantly (2.02 fold, p = 0.20) (Table 3).

In order to determine the specific cellular localization of the p21 and p53 proteins, we conducted immunohistochemical analysis on sections of the right proximal femur of both flight animals and synchronous ground controls. Using a rabbit anti-mouse p21 polyclonal antibody, we found that osteoblasts along the periosteal surface of the proximal femur were p21-positive in flight samples but not in synchronous ground controls (n = 6, Fig. 6A–B). Muscle fibers surrounding the femur contained strongly positive p21 staining nuclei in spaceflight but not ground control samples (Fig. 6C–F). When we investigated the expression of p53 in proximal sections of the femur using a p53 polyclonal antibody, no significant staining was found in most sections from both flight and ground controls’, indicating that p53 was not strongly expressed.

### Spaceflight Alters Gene Expression That Can Interfere with Survival and Mitogenic Signal Transduction

As cell signaling promoting cell survival via phosphoinositide 3-kinase (Pi3K)/protein kinase B (Akt) and cell proliferation via Mitogen Activated Protein Kinase (MAP kinase) can both be activated in response to mechano-transduction, and as they can modulate p53 and p21 signaling, we investigated key genes of interest in these pathways with qPCR. MAP kinases, such as MAPK10, MAPK11, and MAPK12 (3.81, −1.54, and −1.43 fold respectively, p<0.01), MAP 3 kinases, such as Moloney sarcoma oncogene, Mos (−4.18 fold, p<0.01), and cell growth molecules including Egr1 (−2.30 fold, p<0.01) showed altered expression in spaceflight (Fig. 5, Table 3). Several molecules associated with Ras signaling displayed alterations, including Nf1 (−1.45 fold, p<0.05) and Sos1 (−2.51 fold, p<0.01); however, only small changes in KRas expression was found (−1.24 fold, p<0.05) (Fig. 5B, Table 3). No statistically significant alterations in Pi3K subunits were noted except a small change in the alpha polypeptide of the catalytic subunit of Pi3K (−1.39 fold, p<0.01) (Table 3). Additionally, no statistically significant alterations in Akt1 or 2 were found, however, a small change in Akt3/PKB was observed (−1.32 fold, p<0.01) (Fig. 5B, Table 3). After exposure to microgravity, the NF-κB inhibitor, IκBα, exhibited a significant increase in expression (2.03 fold, p<0.01) and NF-κB mRNA exhibited a slight decrease in expression (−1.27 fold, p<0.05) (Fig. 5B, Table 3). Several downstream effectors of these signaling pathways were also altered in response to spaceflight, including Rps6kb1, a down-stream effector of the Pi3K/Akt/mTOR pathway, and Eif4ebp1, a repressor of translation (−2.05 and 1.55 fold respectively, p<0.05). Eif4e, which is activated by Rps6kb1 and inactivated by Eif4bp1, was also down-regulated slightly by spaceflight (−1.40, p<0.05) (Fig. 5B, Table 3). Transcription factors and transcriptional targets associated with these pathways were also investigated and it was found that activated transcription factors c-Fos and Nfatc4 (−2.37 and −1.98 fold respectively, p<0.05) were altered in response to spaceflight. Analysis of NFκB transcriptional target genes involved in cell adhesion, regulation of apoptosis and growth factors and ligands including Traf1, BMP4, Egr1, p53, and MMP3 (repressor) were found to be down-regulated, indicating inhibition of this pathway after exposure to spaceflight (Fig. 5B, Table 3).

## Discussion

In this study, we investigated cellular and molecular gene expression mechanisms underlying bone degeneration in space, with an emphasis on the processes related to osteoblastic and osteocytic activities in the pelvic girdle and proximal femur of 16-week-old C57BL/6J female mice. Although data in this field is not extensive, exposure to microgravity is known to alter bone homeostasis, causing primarily increased osteoclast-mediated bone resorption, and possibly inhibition of bone formation. Here we sought to test the hypothesis that like osteoclastic activity, osteogenic cell cycle arrest and increased osteocytic osteolysis, may also contribute to bone loss in space.

We first characterized standard bone mineral and structural parameters following spaceflight to establish the magnitude of bone loss in our microgravity model system. Our results confirm the ischium portion of the pelvis (Fig. 1A) lost 6.3% (p = 0.005) bone volume fraction and 11.9% (p = 0.002) in bone thickness (Table 1) with an accompanying reduction in trabecular bridges between ischial surfaces (Fig. 1E–G). In addition, bone loss was accompanied by large increases in osteoclast numbers (197%) and osteoclast-covered trabecular bone surfaces measured in the femoral head (154%) (Fig. 3A–D). This observed initial (15-day) rate of bone loss, translated to approximately 12%–24% loss per month, is high compared to established long-term astronaut bone loss values of 1–2% per month. The high-levels of osteoclastic activity we observed may, however, subside after the initial transition to microgravity. On earth at 1 g, patients with tetraplegia show an initial phase of pronounced cancellous bone loss, up to 30%, that subsides one-year post onset of paralysis [37]. Bed rest also results in increased osteoclast activity within 24 h of immobilization [38], and 120 d bed rest causes increased bone resorption surfaces in the pelvis by osteoclasts [39]. After this initial phase of osteoclastic resorption in patients, cortical bone loss continues, although at a reduced rate that is medically more manageable. If osteoclastic activity returns to normal after a period of transition after unloading, continued bone loss may correspond to reduced osteogenic activity and osteocytic osteolysis. Finally, the geometry of the ischium in flight samples is also altered with a wider marrow cavity conformation than ground controls (Fig. 1B–D, Table 2). Specifically, bone geometry near the ischial tuberosity, which provides a hindlimb muscle attachment surface, appears most altered. Among the muscles attaching to the ischium, the quadratus femoris plays an important “antigravity” role in maintaining a neutral hind-limb posture and can atrophy in microgravity [40], possibly explaining the high levels of bone loss we observed in the pelvis.

Having established that microgravity induces high-levels of osteoclast-mediated bone resorption in our model system, we then sought to determine if osteocytic osteolysis was also activated. Osteocytes, are the most abundant cell type in bone and are thought to play a role in spaceflight-induced bone loss [41], [42], [43]. Lacunar enlargement was previously reported in a 22-day spaceflight in the 1970s with juvenile rats but was not conclusively demonstrated [34]. However, mice subjected to hindlimb unloading do not show increased lacunar area, suggesting that osteocytic osteolysis does not play a role in bone loss due to hindlimb unloading at 1 g [36]. Using nanoCT (Fig. 2), we found no bone density differences in non-lacunar regions between flight and ground samples. However, flight samples did exhibit lacunar enlargement relative to ground controls, as well as decreased circularity, and increased canalicular diameter (Fig. 2C–G). These results suggest an increase in osteocytic degradation of lacunar surfaces and highlight a possible difference in the ground-based model for mechanical hindlimb unloading at 1 g versus unloading in microgravity. Furthermore, the number of TRAP-positive osteocytes in flight samples was increased compared to ground controls, with no apparent differences in cell death or lacunar emptying (Fig. 3E–H). Although TRAP is an iron containing enzyme known to be a specific and highly sensitive marker of bone resorption by osteoclasts, Nakano *et al* (2004) also showed TRAP activity in osteocytes [44]. TRAP-active osteocytes in the trabecular and cortical bone may therefore contribute to bone resorption in lacunae [44]. Furthermore, Qing *et al* (2012) found that gene expression analysis of osteocyte-enriched bone from lactating mice exhibited increased expression of TRAP, as well as several other osteoclast specific genes such as cathepsin K, carbonic anhydrase 1 and 2, and MMP13 [36]. TRAP may also be needed for normal bone mineral remodeling and endochondral ossification, as mice lacking TRAP exhibit an osteopetrotic phenotype [45]. It is currently debated whether osteocytes contribute to bone remodeling and loss during spaceflight (for full review see [33]), however, our results support a role for microgravity-induced osteocytic osteolysis.

To further investigate osteocyte-mediated molecular mechanisms associated with bone loss in space, we analyzed gene expression changes relevant to bone extracellular matrix in marrow-free bone tissue containing predominantly osteocytes but also osteoblasts. We found increases in mRNA levels for several collagen molecules and laminin subunits, suggesting increased matrix remodeling, and significant up-regulation of mRNA for matrix metalloproteinases 1, 3, and 10 in flight samples compared to ground controls, while MMP inhibitors showed no statistically significant alterations in expression (Fig. 4, Table 3). Remarkably we also found that osteocytes embedded within the cortical bone of the proximal femur were strongly MMP10 positive in flight samples but not in ground controls, indicating that the up-regulation of MMP10 transcripts in flight samples may originate in osteocytes. ECM proteinases play a critical role in normal pericellular matrix remodeling following environmental stimuli, such as mechanical load, and matrix calcium regulation in normal physiological states as well as in many disease states, such as rheumatoid arthritis and osteoarthritis [46], [47], [48]. Previous studies have shown that osteoclasts secrete MMP1 and 3 during bone remodeling [49]. Furthermore, stromelysins (MMP3 and 10) retain significant activity in the acidic environment found in osteoclast resorptive pits and are secreted by osteoclasts in the sub-osteoclastic resorptive zone [49]. The number of dendritic processes from an osteocyte also increases with age, indicating that osteocytes embedded within the mineralized matrix may have the ability to generate new processes [50], [51]. In total, this gene and protein expression data, together with the ability of osteocytes to express the osteoclast marker TRAP and MMP10, support a role for osteocytic osteolysis in microgravity.

In addition to a role for osteocytic osteolysis, we also hypothesized that bone loss in space may be mediated by intracellular signaling cascades that result in activation of p53 and p21 during osteogenesis. Tumor suppressor protein 53 (p53) is a growth suppressor and a transcriptional regulator which modulates the expression of a wide range of genes involved in cell cycle arrest, apoptosis, and differentiation [52] and p53 activation generally results in apoptosis. Mice null for p53 exhibit extensive blood tumors and an osteopetrotic bone phenotype, indicating a role for the p53 signaling pathway in the regulation of bone formation and degradation [29], [30]. Previous studies have also demonstrated that p53 levels are increased in the muscle of animals exposed to spaceflight [27] and that p53 knockout mice have preserved trabecular bone volume following hindlimb unloading [28]. In contrast to these findings we determined that mRNA expression levels of p53 in marrow free bone tissue was down-regulated and immunohistochemical analysis showed no nuclear or cytoplasmic accumulation of p53. Furthermore, examination of osteocyte nuclei in lacunae from cortical bone of the femur also revealed no differences in the number of empty lacunae, or nuclei with pyknotic morphology in spaceflight versus ground control animals. Previous research, however, has found that hindlimb unloading can result in osteocyte apoptosis and bone loss in the lumbar region of the spine [41]. Research into spaceflight versus 1 g simulations of spaceflight has, however, found differences in bone loss mechanisms including in trabecular bone loss, cancellous mineralization, and bone resorption rates [53]. Our data, including decreased message levels of p53, lack of protein accumulation of p53, and no alterations in osteocytic cell death, suggests that in contrast to hindlimb unloading, osteocyte apoptosis is mostly absent in short-term spaceflight.

Previous studies have shown that periosteal bone formation is inhibited [25] and osteoblast populations are decreased [24] in response to spaceflight, however, the molecular mechanisms causing this arrest of bone formation are not yet known. In contrast to p53, which we found to be down-regulated, expression of CDKN1a/p21, a cell cycle arrest mediator immediately downstream of p53, and other stress response pathways were up-regulated during spaceflight (Fig. 6, Table 3), which may cause an inhibition of osteogenesis and bone formation. Immunohistochemical analysis showed that p21 in flight samples was strongly localized to osteoblast nuclei on the periosteal surface of the femur and near the femoral head. In addition, spaceflight animals showed increased nuclear p21 staining in muscle fibers attached to the femur. MRL mice, which regenerate cartilage, skin, hair follicles, and myocardium with high fidelity and without scarring [54], also lack p21 expression, providing evidence that p21 could play a role in inhibiting tissue regeneration. In addition, CDKN1a/p21 knockout mice also have a regenerative phenotype similar to MRL mice, with normal development, early entry into S phase of the cell cycle, enhanced proliferation, and sensitivity to UV-induced apoptosis [31]. Elevated CDKN1a/p21 levels in spaceflight may therefore explain the observed reduction in osteogenesis and bone growth. The expression of CDKN1a/p21 may also occur in tissues, possibly resulting in a systemic cell cycle and tissue regenerative response. In addition to bone, our gene expression results also show accumulation of p21 in muscle (Fig. 6) and heart tissue (A. Kumar, E.A.C. Almeida, and R.K. Globus, unpublished results). One possible mechanism for systemic expression of CDKN1a/p21 in the mouse may be oxidative stress induced by reactive oxygen species (ROS). Elevated levels of ROS can result in increased lipid peroxidation and decreased levels of antioxidants, which have been reported both in spaceflight and hindlimb-unloading studies [55], [56], [57], [58]. Although the effect of CDKN1a/p21 accumulation in muscle and cardiomyocytes is not addressed in this study, it is possible that a systemic activation of CDKN1a/p21 by ROS could be occurring and might interfere with generalized tissue proliferation, repair and regeneration.

Various other genes associated with cell cycle regulation were altered in response to spaceflight, including cell cycle arrest molecules and cell cycle regulation molecules (Table 3). Such molecules included up-regulation of Fbxo4 and Fbxo31 that have been shown to stimulate the ubiquitination of Ccnd1 [59], [60], [61]. Ccnd1 promotes the transition from G1 to S phase of the cell cycle and is also considered a mediator of signaling from extracellular stimuli to the cell cycle machinery. Furthermore, several of the up-regulated molecules, specifically, Ube2e3 and Mul1, are thought to be involved in cell growth arrest due to mitochondrial fragmentation. Although we have found no cellular evidence of increased apoptosis, several apoptosis-related genes were also up-regulated in flight samples including Cradd, Dapk1, Fbxo31, Hecw2 and MAPK10 (Table 3). The expression of these genes without increased cellular indications of apoptosis could be related to the survival-promoting function of p21 when the cell cycle is arrested.

In order to determine the influence of spaceflight on osteogenic signaling pathways, we conducted RT-qPCR analysis of growth factor transcripts for genes involved in osteogenesis, bone mineralization, and their regulatory transcription factors. Altered growth factor binding in particular, can have significant impacts on intracellular signaling cascades and gene expression. We found message expression reductions in TGF-β2; FGF1 and 3; BMP4; Egr1; and Csf3 (Fig. 5, Table 3). Tgf-β is produced by osteoblasts and deposited in bone matrix during bone formation, and released by osteoclasts during bone resorption [62]. Additionally, Tgf-β induces proliferation and differentiation in osteoblasts while inhibiting osteoclast precursor differentiation and bone resorption [63], [64]. Decreased levels of Tgf-β in the hindlimb of flight samples compared to ground controls has previously been reported. Tgf-β has also been suggested to play a critical role in the mechanosensing ability of bone and bone cells by serving as an intracellular messenger between strain responsive cells and other bone cells [65]. Decreased levels of Tgf-β in the periosteum but not in the cancellous compartment of the hindlimb, correlated with decreases in periosteal bone formation and mineral deposition rates, are suggestive of a role for Tgf-β in mechanical-loading-induced bone formation.

We also found decreases in the expression levels of Bmp4 message (Table 3), which is one of the most potent inducers of bone formation through stimulation of osteoblast differentiation. It has recently been shown that Bmp4-induced cell cycle arrest during osteoblast differentiation is dependent on the induction of p21 and p27 expression [66]. Several additional genes associated with cell growth and differentiation including Myod1, Twist1, and Egr1 were also altered in spaceflight (Fig. 5A, Table 3). Myod1 is known for its function in muscle cells where it causes increased differentiation through activation of p21 [67]. Myod1, however, is also broadly expressed in other tissues and down-regulation of Myod1 message is suggestive of decreased p21-mediated cell differentiation. In agreement with this, Twist 1, known to be involved in differentiation by inhibiting DNA binding by Myod1 and transactivation by Mef2, was up-regulated. Decreased Myod1 expression suggests a decrease in CDKN1a/p21 levels associated with cell differentiation, and leads us to hypothesize that the CDKN1a/p21 elevation we observe in spaceflight is independent of differentiation, and possibly induced by the oxidative stress related to reduced mechanical load in microgravity. Egr1 has been shown to be regulated by c-Fos [68], however, Egr1 activation has also been shown to occur independently of c-Fos through activation of the Erk 1/2 pathway in response to shear stress. Although the exact mechanism regulating Egr1 activity is not examined here, down-regulation of this molecule has important implications for cell growth and differentiation due to its potential role in the regulation of over 30 genes. Furthermore, decreased levels of Egr1 leads to decreased formation of the Egr1/Sp1 complex resulting in increased levels of free Sp1 available for binding to the m-CSF promoter, increased m-CSF expression, and consequently, more osteoclast formation [69]. The observed decreases in TGF-β message could also contribute to decreased Egr-1 expression.

Vitamin D receptor and Sox9, two transcription factors involved in osteogenesis, were also differentially expressed in spaceflight. Vdr gene expression is stimulated by Ras/Raf/MAP kinase signaling through the AP-1 site on the Vdr promoter [46], [70]. Vitamin D has been shown to modulate NFκB activity in multiple cell types, which suggests a role for the Vitamin D receptor in regulation of the NFκB pathway. Vdr-null mice have been shown to have decreased IκBα levels due to loss of vdr-p65 interaction affecting NFκB transcriptional activity [48]. This could provide an additional mechanism for the observed inhibition of NFκB activation in our spaceflight samples. The MAP kinase-ERK pathway is also known to activate the transcription factor Sox9 [71], which is a regulatory factor required for chondrocyte differentiation [72]. Activation of Sox9 by p38-MAP kinase signaling results in chondrocyte growth and differentiation while depression of Sox9 transcription results in cartilage degeneration and osteo-arthritis [55], [73]. Additionally, Sox9 is important for extracellular matrix production [73]. P38-MAP kinase down-regulation and loss of Sox9 transcriptional activation may therefore have important implications for cartilage health and regeneration, and matrix production in spaceflight. Rps6kb1, a down-stream effector of the Pi3K/Akt/mTOR pathway, was also down-regulated in spaceflight (Table 3), and is involved in cell proliferation through the activation of protein synthesis by Eif4e and inhibition of apoptosis through phosphorylation of BAD (for full review see [56]).

### Conclusions

In total, our findings demonstrate the pelvic-femoral musculo-skeletal complex as a highly load and gravity sensitive structure that can serve as a valuable model to study muscle and bone degeneration in space. Additionally, we demonstrate that bone responses to spaceflight are complex and involve osteoclastic, osteoblastic, and osteocytic degenerative responses. Finally, our findings on CDKN1a/p21 up-regulation in microgravity have the potential to become a major concern for tissue regenerative health in long-duration spaceflight. Future studies should address the specific molecular regulatory mechanisms of CDKN1a/p21 involvement in tissue degeneration during long-term spaceflight and the possibility of systemic activation of this strong molecular inhibitor of regenerative adult stem cell proliferation.

## Methods

### Ethics Statement

All experimental animal procedures for STS-131 were approved by the Institutional Animal Care and Use Committee at the National Aeronautics and Space Administration (NASA) Ames Research Center under protocol NAS-10-002-Y1, and conformed to the U.S. National Institutes of Health Guide for the Care and Use of Laboratory Animals.

### Animals

Female, 16-week-old C57BL/6J mice (n = 8) were subjected to 15-days of spaceflight on board the space shuttle Discovery during the STS-131 mission, and as part of the NASA Biospecimen Sharing Program for the spaceflight experiment “Mouse Antigen-Specific CD4+ T Cell Priming and Memory Response during Spaceflight”. Animals used in this study were not subjected to experimental procedures during spaceflight. Animals were housed in the NASA Animal Enclosure Module (AEM) habitats for the duration of the flight and were exposed to normal day/night cycles. The NASA AEM units are composed of a stainless steel grid cage, fan blowers, a layered filter system, interior lamps, food bars and a water box. They are self-contained habitats that provide up to 8 mice with constant access to food and water while also providing a waste management system that isolates the animals from their waste and food crumbs. The AEMs were housed within a standard middeck locker on board the space shuttle. Animals were not acclimated to habitats prior to flight, but were provided with both the food bars and water bottles used in flight for at least 2 weeks prior to launch. Synchronous ground controls were also conducted using identical AEM modules. Spaceflight and synchronous ground control animals were weighed pre- and post-flight and were subjected to veterinary examination before euthanasia. Food and water consumption of each group was measured upon recovery and averaged per mouse per day. Animals were euthanized approximately 2 h after landing using isoflurane anesthesia followed by thoracotomy. Upon recovery, the pelvis and femur were dissected and the soft tissue was removed from the bones. The bone marrow was flushed from both left and right ilium and proximal femora and used for other experimental procedures not described in this paper. Care was taken to avoid disturbing the cancellous bone and bone marrow in both femoral heads during aspiration, to allow for TRAP and immunohistochemical analysis of this region. Both ilia were placed in 1 ml RNAlater at 4°C for 24 h to enable it to penetrate the bone. Vials were then transferred to −80°C and shipped on dry ice to NASA Ames Research Center for isolation of RNA and gene expression analysis. The pelvic ischium and proximal femur were fixed in 4% paraformaldehyde for 24 h at 4°C, washed twice with PBS, and stored in PBS at 4°C. The pelvic ischium was used for microcomputed tomography (µCT) and nano-computed tomography (nCT), while the proximal femur was used for TRAP staining and immunohistochemical analysis of proteins of interest as described below.

### Micro-Computed Tomography (µCT)

Micro-computed tomography (µCT) was used to image and quantify bone morphometry of the ischium region in the right coxa (SkyScan 1174 microCT scanner, Kontich, Belgium). Of the original 8 samples in each group, both flight and ground control had one sample that was fractured during the dissection rendering it unsuitable for the µCT analysis (n = 7).

#### Scanning

Each ischium with soft tissue still intact, previously fixed in 4% paraformaldehyde and stored in PBS, was mounted vertically into a low-x-ray density Styrofoam holder, wrapped with Press-n-Seal film to prevent drying, and scanned in air. Images were acquired at 50 kV and 800 µA with 0.5 mm aluminum x-ray detector filter, a pixel resolution of 6.77 µm, voxel volume of 310.29 µm^3^, an exposure time of 3.5 s per frame with 3 averaging frames, a rotation step of 0.5°, and a rotational angle of 180°.

#### Reconstruction

Raw image data were reconstructed into a stack of 2D cross-sectional slices using NRecon volumetric reconstruction software (Skyscan v1.6.3.2). Reconstruction was carried out with a beam hardening correction of 30%, a ring artefact correction of 4, and a dynamic contrast range of 0 to 0.10.

#### Analysis of bone structure and mineral density

For analysis, a selected region of 4.89 mm (720 slices), starting from 5 slices proximal to caudal apex of the obturator foramen through ischial tuberosity (Fig. 1A), was analyzed using CTan software (Skyscan v1.10.9.0). A global binarization threshold of 65/255 was applied to the reconstructed images. As the mouse ischium is a thin bone with similarly sized bridge-like trabeculae connecting two opposing cortical sheets, we were unable to analyse cortical and trabecular bone regions separately. Instead we contoured along a perimeter of the cortical bone using a “shrink-wrap” custom plug-in of CTan software and analyzed them together. The following indices for mineralized tissue were investigated: total volume of region of interest (TV), bone volume of region of interest (BV), bone volume fraction (BV/TV), average thickness (Th), and mean linear attenuation coefficient. In addition to calculated indices, 3D analysis included the generation of color-coded image maps of ischial structure thickness. These images were loaded into CTvox 3D visualization program (Skyscan v1.0.or479) for a qualitative comparison of bone thickness between flight and ground control groups. To gain some insight on whether the shape of the ischium had changed with the flight environment, we examined a single cross-sectional slice, cut at the dorsal-most point of the descending ischial ramus, and measured the bend angle, the length, and the width at two points, half and one-third of the way from the proximal end (Fig. 1D), using CTan measurement tools. A two-tailed student’s t-test (GraphPad PRISM) was carried out to compare the means, with p-value <0.05 considered to be statistically significant.

### Nano-Computed Tomography (NanoCT)

Following spaceflight, we performed high-resolution measurements of bone density and lacunar nanostructure using the hard X-ray Transmission Microscopy (TXM) facility at the Stanford Synchrotron Radiation Light Source (SSRL). Analysis was performed on similarly oriented transverse sections of cortical bone from mouse ischia near the dorsal-most point of the descending ischial ramus.

#### Sample preparation

Ischia from seven ground control and eight spaceflight mice were fixed in 4% paraformaldehyde and stored in PBS, prior to cutting 30 µm thick cortical bone sections using single-use steel histology blades.

#### Scanning

X-ray attenuation images were acquired at 5.4 keV energy with 40 nm resolution. Triplicate 0° angle images of each sample for osteocyte lacunar and canalicular analysis were obtained with an exposure of 4 s and a beam intensity of 2. Images at 90°, were exposed for 8 s with a beam intensity of 1, and used to measure section thickness.

#### Measurement of bone mineral density

For calibration of mineral density to an X-ray attenuation curve, we used chlorapatite crystals standards and methods previously described [22]. Briefly, to account for beam intensity variations, for each synchrotron experimental run we imaged known size and mineral density chlorapatite crystal standards and plotted log x-ray attenuation relative to thicknesses in the 1 to 100 m size calibration range. Bone density was then calculated from measured 0° bone attenuation values and 90° section thickness values, relative to the standard x-ray attenuation curve.

#### Lacunar morphometry

For quantification of lacunar area, circularity, and perimeter, we used Image J software to trace lacunae in 0° angle images and measured area, circularity, and perimeter in the outlined regions of interest. To measure changes in the dimensions of canaliculi following spaceflight, we selected a 15 µm region of interest extending into the bone from the lacunar wall surface, and measured canalicular diameters. Unpaired t-test analysis was carried out to compare the means, with p-value <0.05 considered to be statistically significant.

### Tartrate Resistant Acid Phosphatase (TRAP) Staining

The right proximal femur was used for tartrate resistance acid phosphatase staining. Bone marrow was gently aspirated from the shaft region of the femur so as not to disturb cancellous bone and bone marrow in the femoral head. Flushed bone was then demineralized using 20% EDTA for 21 days, with solution changes every three days. Decalcified bones were dehydrated in a graded ethanol series and embedded in paraffin in preparation for immunohistochemical analysis. Longitudinal sections of 7 μm thickness were prepared and immediately before analysis, sections were deparaffinized in 100% xylene, and rehydrated in 100% EtOH, 95% EtOH, 75% EtOH and DI H_2_O consecutively. TRAP staining was then conducted on these sections (n = 6 sections per animal, n = 5 mice). The sections were stained for tartrate resistant acid phosphatase (TRAP) using an Acid Phosphatase Leukocyte Kit (Sigma-Aldrich, Catalog #387A-1KT) according to the manufacturer protocol. Images were acquired immediately after staining at magnifications of 20× and 40× using a SPOT RT slider cooled CCD camera attached to an Olympus BX51 microscope. All images were taken at the same balance, gain, and brightness. To quantify osteoclast numbers and osteoclast contact with bone surfaces we imaged the central cancellous bone region in the proximal femur just below the growth plate. Criteria for inclusion of osteoclasts in cell counts included TRAP-positive staining, at least 4 nuclei, and a contact surface with cancellous bone of at least 10 µm. Using Image J, we measured the total number of osteoclasts that met the inclusion criteria, length of cancellous bone surface, and length osteoclast contact surface with cancellous bone. The following indices for mineralized tissue were investigated: total number of osteoclasts per mm of bone surface (N.Oc./BS), and percentage of osteoclast-covered surface (Oc.S./BS). To analyze osteocytes, Image J was used to perform a cell count of TRAP-positive osteocytes and total number of osteocytes in the cortical bone immediately distal to the growth plate of the femur. All statistical analyses were performed using an unpaired t-test to determine the significant differences between ground control and spaceflight samples.

### RNA Isolation and RT-qPCR Analysis

Total RNA was isolated from both RNAlater-fixed pelvic ilia (flushed of bone marrow) from spaceflight and ground control animals using the TRIzol method (Invitrogen). Briefly, ilia were homogenized with a hand held homogenizer in 1 ml of TRIzol reagent (Invitrogen, Catalog #15596-026) for 30–60 s. Homogenized samples were incubated in TRIzol for an additional 5 min, 200 μl of chloroform was added and samples were incubated for a further 3 min. Samples were centrifuged at 12,000 g for 15 min at 4°C. The aqueous phase containing RNA was placed into a new tube and RNA was precipitated with 600 μl of isopropanol for 10 min. Samples were centrifuged again at 12,000 g for 10 min at 4°C and the supernatant was discarded. RNA was washed with 1 ml 75% EtOH, centrifuged at 7,500 g for 5 min at 4°C, the pellet was air dried and re-dissolved in 30 μl RNase-free water. Samples were then purified using RNeasy mini kit (Qiagen, Catalog #74104) with genomic DNA elimination step according to the manufacturers protocol. RNA concentration was measured using spectrophotometry (Nanodrop, ThermoFisher) and quality was examined with agarose gel electrophoresis. Gene expression was analyzed using Qiagen Pathway-Focused Mouse qPCR Arrays. We examined genes involved in osteogenesis, extracellular matrix, ubiquitination, and the p53, Pi3K, NFκB, and MAP Kinase, signaling pathways in bone tissue. Each array consisted of primer sets for 84 pathway-focused genes of interest, five reference genes (Gusb, Hprt1, Hsp90ab1, Gapdh, and Actb), one genomic DNA contamination control, three positive PCR controls and three positive reverse transcription controls on a 96 well plate. One μg of total RNA per sample (n = 6) was reverse transcribed to cDNA using RT^2^ First Strand cDNA Synthesis Kit (SABiosciences, Catalog #330401) according to the manufacturer’s protocol. Briefly, genomic DNA elimination buffer was added to the sample and incubated at 42°C for 5 min. Reverse transcription cocktail containing primers, external controls and RT enzyme mix was then added to the samples and samples were incubated at 42°C for 15 min and the reaction was stopped by incubation at 95°C for 5 min. The cDNA was mixed with RT^2^ SYBR Green/Rox qPCR master mix and equal volumes (25 μl) were added to PCR plates. Plates were sealed with optical thin-walled 8-cap strips (Qiagen, Catalog #1065667) and qPCR of sample arrays was performed using an Applied Biosystems 7500 Real Time PCR instrument. Real-time PCR conditions were as follows: one cycle 95°C for 10 min, 40 cycles of 95°C for 15 s and 60°C for 1 min, followed by one cycle of 95°C for 15 s, 60°C for 1 min, 95°C for 15 s and 60°C for 15 s. Gene expression levels from all arrays were analyzed for alterations in expression levels as compared to ground controls (n = 6, p<0.05) using a PCR Array Data Analysis Template (v3.2, SABiosciences). Data analysis was based on the ΔΔCt method and gene expression levels were normalized to four reference genes (Gusb, Hprt1, Gapdh, and Actb).

### Immunohistochemical and Immunocytochemical Analysis

Sections of the right-proximal femur prepared as described above for TRAP Staining were also used for immunohistochemical and immunocytochemical analysis. The following primary polyclonal antibodies were used in the immunohistocehmical analysis: anti-p21 (1∶50 dilution, Santa Cruz Biotechnology), anti-p53 (1∶50 dilution, Santa-Cruz Biotechnology), anti-Matrix Metallopeptidase 10 (1∶20 dilution, Novus Biologicals) and anti-Mdm2 (1∶25 dilution, Santa Cruz Biotechnology). Peroxidase conjugated donkey anti-rabbit IgG was used as the secondary antibody (1∶5000 dilution, Jackson Immunoresearch). Briefly, sections were immersed in sodium citrate buffer for 10 min at 95°C, washed in DI water and immersed in PBS containing 0.1% Triton X-100, pH 7.4 (2×5 min). Sections were then blocked in 10% normal blocking serum (Donkey serum in PBS-T) for a period of 1 h in a moist chamber at room temperature. Primary antibody incubation was conducted in a moist chamber at 4°C overnight. Sections were washed in PBS-T and incubated with peroxidase conjugated secondary antibody in a moist chamber at room temperature for 1 h and then rinsed with PBS-T and incubated in peroxidase substrate for 12–30 min until staining was complete. Sections were washed in DI water and dehydrated in 75%, 95% and 100% EtOH followed by 100% xylene for 3 min at each stage. Sections were mounted in mounting medium and visualized using an Olympus BX51 light microscope. For osteocyte nuclear localization, we used Hoechst 33342 staining (500 ng/ml in DI water for 10 min at room temperature, Invitrogen, Catalog #H1399) on deparaffinized sections of the right-proximal femur prepared as described above. Lacunae with and without Hoechst stained nuclei, and nuclear pyknotic morphology were used to quantify osteocyte apoptosis.
